# Characterization of Honey Microbiome Using MALDI-TOF Mass Spectrometry and Physicochemical Study

**DOI:** 10.3390/molecules30061266

**Published:** 2025-03-12

**Authors:** Dominika Błońska, Bogusław Buszewski

**Affiliations:** 1 Department of Environmental Chemistry and Bioanalytics, Faculty of Chemistry, Nicolaus Copernicus University, 87-100 Toruń, Poland; 2 Centre for Modern Interdisciplinary Technologies—BioSep, Nicolaus Copernicus University, 87-100 Toruń, Poland; 3 Prof. Jan Czochralski Kuyavian-Pomeranian Research & Development Centre, Krasińskiego 4, 87-100 Toruń, Poland

**Keywords:** environmental microorganisms, physicochemical properties, bacteria identification, MALDI-TOF/MS method

## Abstract

Honey, a super-saturated solution produced by *Apis mellifera*, is well-known for its historical medicinal uses, as well as culinary applications. Comprising sugars, phenols, enzymes, and more, its complex composition contributes to its medicinal properties. The microbiome, dominated by spore-forming bacteria and yeasts, is also a crucial factor in the health benefit properties of honey. The identification of the microbiome of honeys contributes to a better understanding of their microbial landscape and health-benefit properties and is also relevant to the environmental aspect. Matrix-assisted laser desorption/ionization time-of-flight mass spectrometry (MALDI-TOF MS) is emerging as a key tool for microbial identification, but challenges remain in ensuring accuracy under different conditions. This study focuses on developing optimal conditions for microbial isolation and culture, aiming to balance diversity and avoid negative effects on identification. It further has the objective of evaluating the influence of geographic and botanical factors on the composition and diversity of the honey microbiome.

## 1. Introduction

Honey, a super-saturated solution primarily composed of sugars (mainly glucose and fructose), is a natural sweetener produced by *Apis mellifera*. It is created by converting sucrose from nectar or honeydew in the bee’s digestive tract under the influence of enzymes and formic acid. Historically, honey has served various purposes, from being the first sweetener used by humans to a natural remedy for health issues [1,2,3]. Its diverse composition includes volatile organic compounds, phenols, polyphenols, enzymes, minerals, and vitamins, making it a complex and valuable substance. The use of honey can improve immunity, support brain function, lower blood pressure, or speed up wound healing. Honey also demonstrates metabolic, prebiotic, and cardiovascular benefits, and its biological activity depends mainly on its botanical or geographic origin [4,5]. The color of honey is a significant characteristic, ranging from very light (colorless) to dark amber or even black. This variation depends on factors such as botanical origin, storage conditions, age, and the degree of glucose crystallization. The Pfund scale is commonly used to classify honey color, measuring it in millimeters. This scale ranges from “water white” for the lightest honeys to “dark amber” for the darkest. These properties can help to distinguish different types and also provide information about the authenticity of the honey [6].

An important component of honey is also its microbiome. The physicochemical properties of honey, such as acidity, concentrated sugar, and water content, influence its microbial composition, resulting in a microbiome dominated by spore-forming bacteria and yeast [7,8,9,10]. The primary sources of microbial contamination in honey samples include pollen, the digestive tracts of honeybees, air, and the plant material itself. Additional sources encompass improper human practices, non-sterile equipment, and containers [6,11]. According to research, the most widespread bacteria in honey include the families *Bacillaceae*, *Lactobacillaceae*, *Enterobacteraceae*, *Acetobacteraceae*, *Microbacteriaceae*, and *Bifidobacteriaceae*. *Clostridium botulinum* spores are also identified in some honey samples, but they are often at low levels [12,13,14]. The presence of spore-forming bacteria in honey is crucial for their identification, as endospores display distinct protein expression compared to vegetative cells. Small acid-soluble proteins (SASPs) are integral to endospore formation, and variations in the incubation temperature of *Bacillus* species can alter protein content by influencing the degree of endospore development. Consequently, establishing optimal conditions is essential for the accurate identification of these environmental microorganisms [15,16,17].

Of great importance are also the antibacterial properties, which are mainly due to the presence of hydrogen peroxide, produced by the glucose oxidase present in honey. Certain types of honey also possess antibacterial properties unrelated to hydrogen peroxide, but associated with the microbiome present in them and the production of secondary metabolites by the latter. These metabolites display bactericidal or bacteriostatic effects against a wide range of pathogens, including those linked to hospital-acquired infections or hard-to-heal wounds, such as *Staphylococcus aureus*, *Staphylococcus haemolyticus*, *Escherichia coli*, *Pseudomonas aeruginosa*, *Klebsiella pneumoniae*, and *Enterococcus faecalis*. They are commonly employed as medicinal agents in various therapies and show promise as potential treatments for drug-resistant pathogens [18,19].

The variability in honey composition, influenced by geographical origin and source material, is a subject of scientific interest. Studies, such as those by Buszewski et al. [20], have explored the correlation between physicochemical properties, including the presence of cyclitols and sugar content, providing valuable insights. Researchers have extensively analyzed the mineral and heavy metal content in honey to assess its nutritional value and potential contamination levels [21]. Assessing heavy metal concentrations in honey and other foods is a critical indicator of overall health and safety [22]. Lasić et al. [23] applied inductively coupled plasma mass spectrometry (ICP-MS) to assess the presence of 15 elements in 174 honey samples collected from three distinct geographic regions in Croatia. Their findings revealed that the mineral composition varied according to the honey’s botanical origin. Notably, elevated levels of lead (Pb) and zinc (Zn) in samples from the Pannonian region served as effective markers for determining the geographical provenance of the honey. Pavlin et al. [24] used a combination of ICP-MS and statistical tools to identify the origin of honey by mineral characterization. Their findings indicated that elements such as manganese (Mn), potassium (K), and calcium (Ca) are influenced by the type of pollen present, suggesting a floral source, while levels of sodium (Na), magnesium (Mg) and iron (Fe) could serve as an indicator of geographic origin.

Honey types are classified based on source material, distinguishing between nectar, honeydew, and mixed varieties. The microbial content of honey, including bacteria, yeast, and fungi, further varies based on factors like climate, flora, and apicultural practices. Understanding these regional differences can uncover unique properties, quality, microbial diversity, and potential health benefits [25,26,27]. Beyond its culinary and medicinal uses, honey plays a crucial role in supporting biodiversity through pollination. The decline in honeybee populations poses environmental challenges, affecting global food supply and pricing [28]. Knowledge of the microbiome can also enable the prevention and spread of bee diseases, which is also crucial for the environment due to the greatly reduced numbers of honeybees. Imbalances in the gut microbiota of honey bees can lead to increased disease susceptibility and contribute to colony decline. Therefore, maintaining a stable and diverse gut microbiome is crucial for honey bee immunity and productivity [29].

According to The Food Agriculture Organization of the United Nations, up to 75% of the world’s food supply is dependent on pollinating insects, including honeybees. A reduction in the number and vitality of bees, responsible for pollinating many crops and plants, can affect the availability and price of food, as well as biodiversity [30]. Furthermore, an example of bee related diseases includes the highly infectious European foulbrood (EFB) and American foulbrood (AFB), induced by the bacteria *Melissococcus plutonius* and *Paenibacillus larvae*, respectively [31,32,33]. AFB affects the larval stage of honey bees, resulting in the death of the bee larvae and their decay in the hive. This disease causes the dysfunction of entire hives and often resides asymptomatically as inactive spores. The lack of initial symptoms is a threat, as the disease can spread throughout the apiary without the beekeepers being aware of the problem. The presence of *Paenibacillus larvae* in honey can indicate its contamination with AFB-infected larvae, which can pose a threat to the entire hive or even the whole apiary, as well as to the quality and safety of the honey. In many countries, such as the United States and those belonging to the European Union, when *P. larvae* are present in hives, beekeepers are obliged to take measures to prevent the spread of AFB in the apiary [34,35,36,37].

After accounting for its renowned antibacterial properties and environmental impact, it becomes clear that a comprehensive understanding of the honey microbiome is relevant [38]. Moreover, the diversity of these microbial communities—shaped by physicochemical, geographical, and botanical influences—not only underpins honey’s health benefits but also serves as a potential indicator of its authenticity. Due to the high costs and time requirements associated with traditional molecular techniques, analytical methods like matrix-assisted laser desorption/ionization time-of-flight mass spectrometry (MALDI-TOF/MS) have become instrumental in identifying microorganisms in honey samples efficiently. This method offers rapid, sensitive, and cost-effective microbial identification, making it a valuable tool in clinical microbiology. However, the accuracy of bacterial identification may be influenced by culture conditions, incubation times, and environmental microorganisms producing spores. Moreover, the reference databases for environmental organisms are relatively limited in scope [39,40]. In addition, the MALDI-TOF/MS mass spectrum can tentatively identify probable proteins responsible, for example, for antimicrobial activity (bacteriocins), based on their *m*/*z* ratio [41]. However, confirmation of their identification requires further studies, in particular MS/MS fragmentation.

The present study aims to select the conditions for isolating, culturing, and identifying environmental microorganisms in honey samples from different botanical and geographical origins. For the identification of bacteria and yeast, the MALDI-TOF/MS was used. Additionally, the study seeks to elucidate the relationship between microbiome composition and geographical region, botanical origin, and physicochemical properties of analyzed honey samples. Recognizing the importance of a broad approach, the present study aims to develop suitable conditions for microbial analysis, ensuring the isolation of a diverse array of microorganisms. It is crucial to strike a balance, as these conditions should not have a negative impact and should allow for the rapid and reliable identification of the microbiome. This approach would contribute to a comprehensive understanding of the microorganisms in honey samples.

## 2. Results and Discussion

### 2.1. Isolation and Culture of Bacteria from Honey

The selection of the culture condition, such as a time of cultivation, was necessary due to the slow growth of certain microorganisms, even after several days. However, fresh bacteria samples are required for identification, as a significant proportion of environmental microorganisms produce spores, changing the protein composition and making identification challenging, and often impossible [42]. Therefore, it was necessary to incubate the plates after the reduction culture for a maximum of 12–14 h. For samples cultured longer (16–24 h), no identification was obtained using the MALDI technique for the short protocol Extended Direct Transfer.

The effect of temperature on the identification of various bacterial species (*Bacillus subtilis* subsp. *subtilis*, *Bacillus cereus*, *Bacillus pumilus*) was also observed. Differences were noted in both the number and type of signals obtained, as well as their intensity, when cultured at various temperatures. Some bacterial strains achieved a satisfactory level of identification after cultivation at 25 °C ± 2 °C, while others only at 37 °C ± 2 °C. In the case of *Bacillus subtilis* subsp. *subtilis* significant improvement in identification was noted when incubated at 25 °C (for example, score = 2.12, providing a secure genus identification and probable species identification) compared to 37 °C (score = 1.95, indicating probable genus identification). A similar result was observed in the case of *Bacillus cereus*, in which the score value improved as a result of the incubation temperature of 25 °C (score = 2.20, secure genus identification and probable species identification) than at 37 °C (score = 2.49, highly probable species identification).

**Figure 1:**
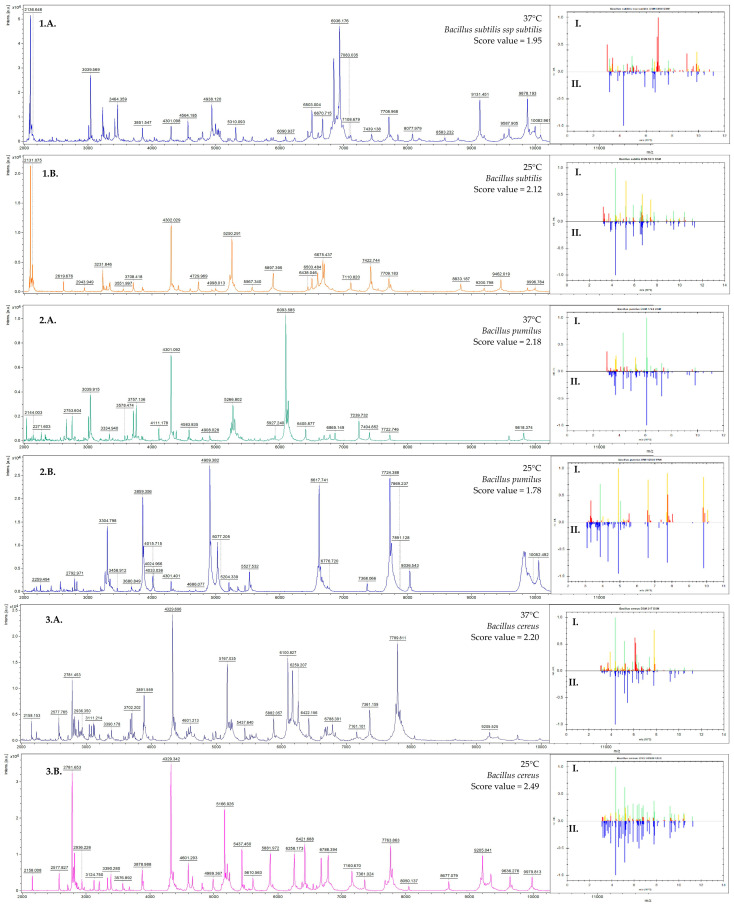
Example spectra of three isolates: *Bacillus subtilus subs. subtilis* (**1.A**.,**1.B.**), *Bacillus pumilus* (**2.A.**,**2.B.**) and *Bacillus cereus* (**3.A.**,**3.B.**) cultivated for 12–14 h in 25 °C (**B.**) and 37 °C (**A.**), obtained from MALDI FlexAnalysis and Biotyper 4.1 software (Bruker Daltonics GmbH, Bremen, Germany). I—obtained MS spectrum compared to II—reference MS spectrum in a database.

Identification results for 83% of the *Bacillus pumilus* strains tested appeared better after incubation at 37 °C (example score = 2.18, secure genus identification and probable species identification) than at 25 °C (score = 1.96, probable genus identification). Longer culture time (<16 h) did not allow for reliable identification results to be obtained. Examples of differences in intensity and the number of characteristic signals on the mass spectra (MS) are illustrated in Figure 1. The level of coincidence between the protein signals of the identified strain (Figure 1(I)) and the reference strain spectrum (Figure 1(II)) can be seen by observing the green signals. In the presented mass spectra, significant differences can be observed in the number and intensity of protein signals obtained (protein profiles) for bacteria incubated at different temperatures.

The effects of culture time and temperature, as well as endospores on the identification of *Bacillus* bacteria have also been described by Shu and Yang [43] and Chambers et al. [44]. The results suggest the control and use of different incubation temperatures for various *Bacillus* bacteria in order to obtain the best possible, reliable identification results using the MALDI-TOF/MS technique. Moreover, it was confirmed that different *Bacillus* species isolated from different environments can have different optimal growth temperatures, which also affects the rate of endospore formation. In addition, it also found an effect of incubation time on the dramatic growth of endospores and simultaneously hindered identification, suggesting the need to use fresh isolates [43].

The observations confirm that bacterial growth temperature significantly impacts phenotypic properties, likely as an immediate response to environmental changes. Moreover, in this process, bacteria adjust cell membrane fluidity by altering phospholipid composition. These phospholipids are responsible for providing a suitable environment for the functioning of membrane proteins; as a result, the change in membrane thickness induced by culture temperature can affect protein activity. Consequently, temperature-induced variations in membrane thickness can affect protein activity, leading to changes in the bacteria’s proteomic profiles [45,46,47,48].

**Figure 2:**
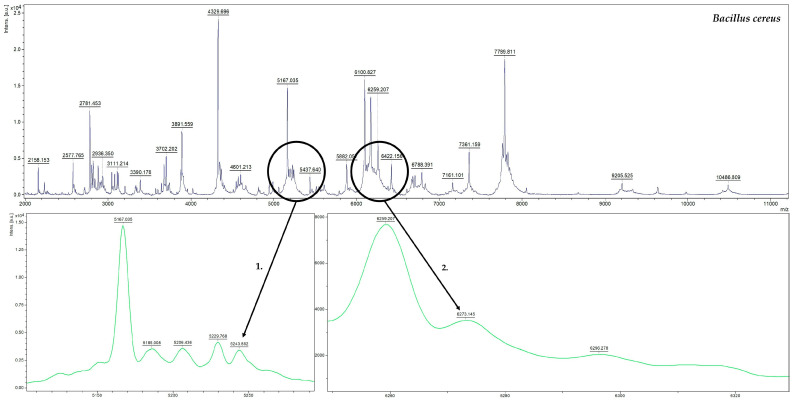
Probable protein signals determined for *Bacillus cereus* strain.

Furthermore, the MALDI-TOF/MS mass spectrum can tentatively identify probable proteins responsible, for example, for antimicrobial activity (bacteriocins), based on their *m*/*z* ratio. Figure 2 shows examples of pre-identified signals for *Bacillus cereus* isolated from honey samples. Two signals were pre-identified from UniProt for the strain: *m*/*z*_1_ = 5243.852 (*m*/*z*_theoretical_ = 5244, mass error = 28.22 ppm) and *m*/*z*_2_ = 6273.145 (*m*/*z*_theoretical_= 6273, mass error= 23.11 ppm). The signal *m*/*z* = 5244 may represent a bacteriocin-type signal sequence domain protein [49], while *m*/*z* = 6273 represents a peptide antibiotic containing lanthionine active on Gram-positive bacteria (lantibiotic) [50]. Bacteriocins are considered safe for use in humans and are used to treat bacterial infections [51]. Lantibiotics are among the most promising alternatives for future antibiotics, exhibiting antibacterial efficacy through multiple mechanisms. Recent studies have identified new modes of action, highlighting their potential for future applications. Ongoing research continues to unveil detailed molecular activities of various antibiotics, offering valuable insights into their function and effectiveness [52]. However, confirmation of identification of protein signals requires further studies, in particular MS/MS fragmentation and in combination with specialized software. While MALDI-TOF MS is powerful for metabolite identification, there is a need for more standardized protocols and comprehensive databases to improve accuracy and reliability [53,54].

### 2.2. Color of Honey Samples and pH Measurements

The color of the honey samples can be affected by various factors, including the content of compounds (such as phenolics, sugars, minerals, pollen, carotenoids, and flavonoids), water content, botanical and geographical origin, storage conditions and duration, as well as the age of the honey. Generally, darker honeys have higher polyphenol levels, while lighter honeys are richer in flavonoids [55]. Honey samples exhibited a wide range of colors, spanning from white (18–34 mm on the Pfund scale) to dark amber (>114 mm). Darker colors, including amber and dark amber (>86 mm), constituted 53% of the samples examined. Notably, 25% of the tested samples displayed a water-white color, measuring <9 mm on the Pfund scale. Intriguingly, one honey sample appeared blue, a result of the presence of spirulina. The measured pH of the honey samples was acidic, ranging from 2.98 to 4.43. The highest pH was recorded in nectar honey, specifically multifloral honey from Radom, Masovian Voivodeship, Poland. Conversely, the lowest pH was observed in another polish nectar honey, acacia honey from Sosnówka, Lower Silesian Voivodeship. The majority of samples were within the accepted range of pH, a crucial parameter, as an increase may indicate honey adulteration [56]. Table 1 shows a list of the investigated honeys along with their type, region of origin, and measured physicochemical parameters (color and pH value).

### 2.3. Bacteria Isolation

As a result of bacteria isolation on five different microbiological media using various temperature conditions and incubation times, 388 bacterial strains were isolated from 73 honey samples. All colonies were then subjected to identification using the MALDI-TOF/MS technique. In seven tested honey samples—Linden (Lower Silesia, Poland), Linden (Podlaskie Voivodeship, Poland), Melilot-cornflower (Pomeranian Voivodeship, Poland), White sweet clover (Subcarpathian Voivodeship, Poland), Linden (Łódź Voivodeship, Poland), Multifloral (Lisbona, Portugal), Chinese acacia and fennel (England)—despite repeating the process twice, no bacteria were isolated.

### 2.4. MALDI-TOF/MS Detection of Isolated Bacteria

**Figure 3:**
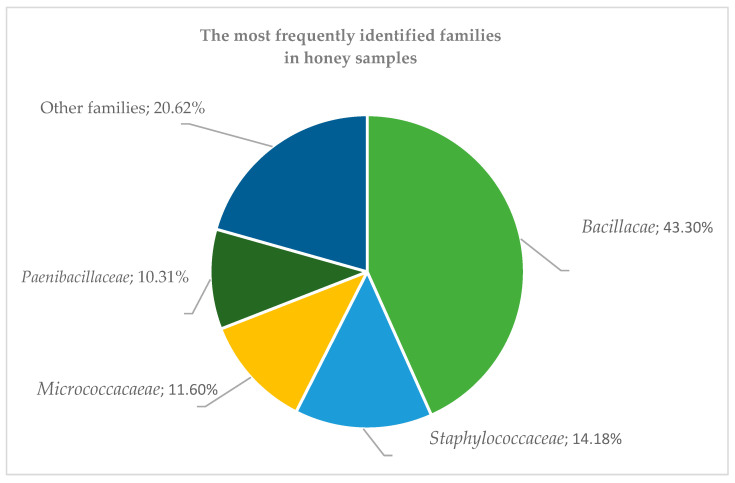
The most frequently identified bacterial families in honey samples.

As a result of microorganism detection by MALDI, 118 strains belonging to 19 different families (and one *Incertae sedis* strain) were identified from the samples. Among these, 97 strains were classified as Gram-positive bacteria, 19 strains as Gram-negative bacteria, and 2 as yeasts. The most frequently identified families were *Bacillaceae* (43.30%), *Staphylococcaceae* (14.18%), *Micrococcaceae* (11.60%), and *Paenibacillaceae* (10.31%). The remaining families accounted for 20.62% of all tested bacteria (Figure 3). The most frequently identified bacteria included *Bacillus pumilus* (present in 36 out of all 73 samples), *Micrococcus luteus* (30 strains), *Ralstonia insidiosa* (26), *Staphylococcus hominis* (25), *Bacillus cereus* (19), *Bacillus subtilis* subsp. *subtilis* (13), *Staphylococcus epidermidis* (12), *Bacillus licheniformis* (10), and *Bacillus megaterium* (9). A shorter incubation time (12–14 h) provided better identification results, increasing the score value.

The high prevalence of *Bacillales*, including the *Bacillus* and *Paenibacillus* genera, in the honey samples aligns with findings reported by other research groups [12,57,58,59,60]. Bacteria from the *Staphylococcaceae* or *Micrococcaceae* families may contaminate honey during production, packaging, or storage, but they typically do not play an active role in its microbial ecosystem. Among the isolated microorganisms, yeasts were also identified, belonging to two species: *Rhodotorula mucilaginosa* and *Starmerella magnoliae.* These species were previously identified as some of the more common in honeys from Portugal [61], as well as in lime honey from Poland [62]. The presence of *Starmerella magnoliae* in honey offers a promising opportunity to isolate wild yeasts capable of producing valuable chemical compounds such as citric acid and mannitol [62]. In addition, the conditions developed allowed the identification of a wide variety of other bacterial species, among which many may exhibit antibacterial properties.

**Figure 4:**
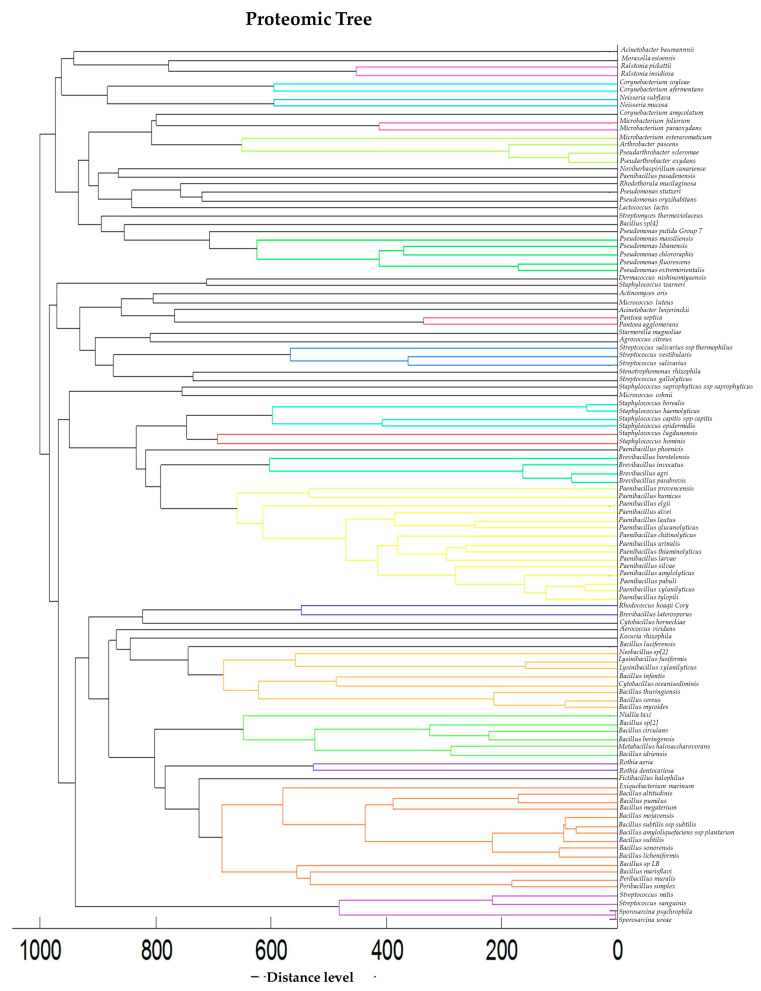
Proteomic tree of identified honey strains.

The microorganisms identified by MALDI were classified as follows: 95 strains achieved probable genus identification (24.48%), 233 strains—secure genus and probable species identification (60.05%), and 60 strains—highly probable species identification (15.46%). Additionally, *Paenibacillus larvae*, the bacterium responsible for American foulbrood—a contagious disease of bee larvae—was identified in four honey samples: Multifloral with spirulina 0.4% (Kuyavian–Pomeranian Voivodeship, Poland), Multifloral (Istanbul, Türkiye), Multifloral (Ankara, Türkiye), Multifloral (Blaj, Romania). While there is generally a low risk of human infection with *Paenibacillus larvae*, the detection of this bacterium in a sample may indicate the presence of disease in the hive. *Paenibacillus larvae* spores can remain infectious for over 35 years in old hives. This complicates disease control, as human activity can facilitate long-distance spread, and dormant strains may trigger outbreaks years after the initial infection [63]. Moreover, *Paenibacillus alvei*, identified in three honey samples, is a common secondary causative agent of European foulbrood [33]. A proteomic tree was prepared to illustrate the relatedness of all 118 identified strains (Figure 4).

### 2.5. Statistical Analysis

To investigate the potential relationships between the presence of specific bacterial strains, pH values, and color in honey samples, as well as the relationship between pH and color, a correlation analysis was conducted using Python 3.12 and the libraries ‘pandas’, ‘matplotlib.pyplot’, and ‘seaborn’. The analysis aimed to explore the correlation between physicochemical properties and the honey microbiome. The main purpose of the analysis was to use Polish samples as a reference, while those from other regions of Europe and the world (N = 28) were included for comparison. However, it should be noted that the number of non-Polish samples was significantly smaller. Therefore, the interpretation of the results should take this limitation into account.

For samples from Poland, the correlation between the number of bacteria and pH was approximately 0.129, indicating a slight positive correlation. This suggests that a higher pH may be slightly associated with a higher number of bacteria, although the relationship is weak. Similarly, for samples from different regions of the world, the correlation was 0.1, showing a comparable weak positive relationship.

Regarding the relationship between pH and color, the correlation was about −0.2720 for both Polish and global samples, indicating a moderate negative association. This implies that lower pH values may be moderately associated with more intense honey color. The similarity in correlation values across different regions suggests that this trend is consistent regardless of the geographic origin of the honey samples. The Pearson correlation matrix was useful in assessing the strength and direction of relationships between variables. These results suggest that pH and color have moderate relationships with certain microbiological characteristics, such as bacterial counts. However, these associations are weak to moderate, indicating that these variables are relatively independent in the context of honey microbiological analysis. The overall weak positive correlation suggests a limited tendency for bacterial numbers to increase with rising pH values. Linear regression analysis did not reveal a strong relationship between bacterial count and honey pH values, except for *Bacillus subtilis* subsp. *subtilis*, where a notable pH effect was observed.

The logistic regression model, which achieved convergence after seven iterations, had an objective function value of 0.3235. The pseudo R-square, approximately 0.3095, indicates a moderate fit of the model to the data. In the logistic regression model, the coefficient for the constant (const) was −23.8901 with a standard error of 6.3350. The coefficient for the ‘pH’ variable was 6.3159 with a standard error of 1.7620. The ‘pH’ variable was statistically significant (P > |z| < 0.001, below the 0.05 significance level), indicating a meaningful relationship. Specifically, the logistic regression model suggests a statistically significant association between *Bacillus subtilis* subsp. *subtilis* presence and pH values. The positive coefficient for pH = 6.3159 implies that an increase in pH values corresponds to an increased probability of isolating *Bacillus subtilis* subsp. *subtilis.* The results of Student’s *t*-test for comparing the means of two independent groups of samples (Polish and non-Polish) in terms of the average number of bacteria showed that the t-statistic was −0.7841. This negative value suggests that the average number of bacteria in Polish samples may be lower than in non-Polish samples, but the difference is insignificant or very small. The *p*-value, which was 0.4356, is greater than the accepted significance level of 0.05, indicating insufficient evidence to reject the null hypothesis.

**Figure 5:**
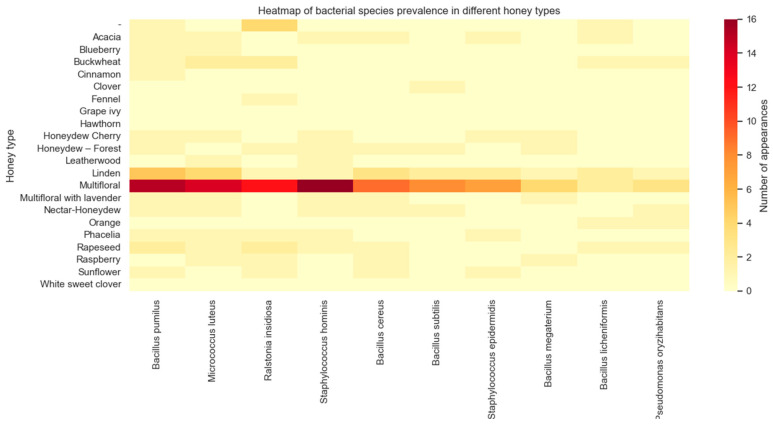
Bacteria species prevalence in different honey types (botanical origin).

Therefore, there is no statistically significant difference in the average number of bacteria between Polish and non-Polish samples. In conclusion, these results indicate that based on Student’s *t*-test, there is insufficient evidence to reject the null hypothesis of no difference in the mean number of bacteria between samples from Poland and samples from outside Poland. Examining the relationship between honey type (botanical origin) and the presence of bacterial species, the chi-square statistic was 3008.73, and the *p*-value was very low (4.3832 × 10^−8^). This indicates a statistically significant relationship between honey type and the presence of bacterial species. The low *p*-value suggests that the differences in bacterial species numbers between different honey types are significant and not due to chance. The significant relationship between honey type and the presence of bacterial species suggests that different types of honey have distinct bacterial profiles. This may be due to specific environmental conditions and production methods associated with each honey type. The botanical origin of honey plays a significant role in its antimicrobial properties, which in turn affect the number of bacteria and other microorganisms (yeast, fungi) that can survive in it. Different plants contribute unique compounds to honey, such as phenolic acids, flavonoids, and hydrogen peroxide, all of which have varying degrees of antibacterial activity. These compounds can inhibit the growth or kill certain bacteria, thereby influencing the overall microbiome and limiting bacterial growth. As a result, honey from different botanical origins may exhibit varying levels of antibacterial effectiveness, which ultimately determines the number of microorganisms that can grow in it [64,65]. To visualize the results, a heat map was created showing the frequency of selected microorganisms in different types of honey (Figure 5).

## 3. Materials and Methods

### 3.1. Collection of Honey Samples

**Figure 6:**
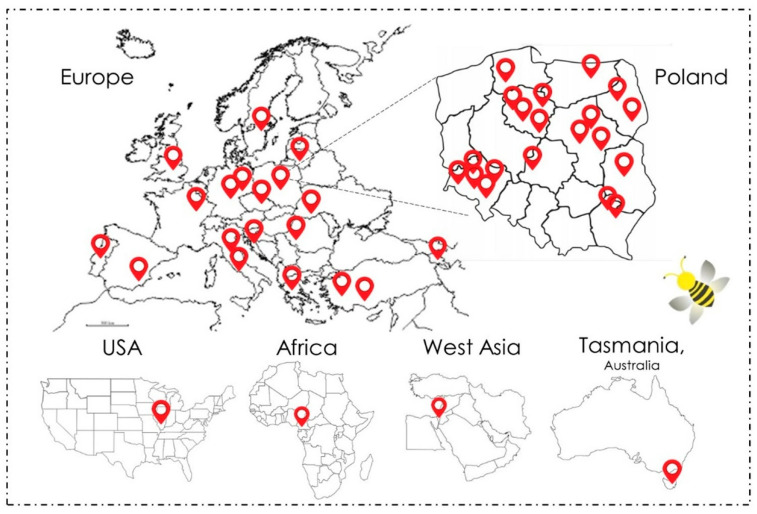
Map presenting the origins of honey samples.

Prior to the study, 73 honey samples were collected from apiaries in different geographical and botanical regions. Most samples were from Poland (45), and others were from various countries worldwide: Germany (5), Türkiye (3), Italy (2), Tasmania (2), and Armenia (2). One sample each was obtained from Greece, Romania, Croatia, Cameroon, the United States of America, France, Portugal, England, Israel, Lithuania, Sweden, Czechia, Spain, and Ukraine (Figure 6). Samples from outside Poland amounted to 28 honeys. The honeys collected varied in botanical origin: nectar honeys (62), honeydew honeys (2), nectar–honeydew honeys (1), and unknown (8). Both nectar and honeydew honeys were derived from a variety of plants as raw materials and were collected in an effort to provide a wide variety of samples. Isolating the greatest possible number of different species can provide an indication of potential antimicrobial activity based on and related to the presence of the tested bacteria in the samples. Honey samples were collected under sterile conditions or obtained in ready-made containers, typically glass or plastic.

### 3.2. Conditions for Bacterial Isolation and Cultivation from Honey Samples

The expected environmental species are from *Bacillus* spp., *Streptococcus*, *Staphylococcus*, LAB (lactic acid bacteria), *Enterobacteriaceae*, and yeasts. To ensure optimal growth and selection, both selective and universal culture media were utilized. Approximately 0.5 mL of honey was added to 4.5 mL of ultra-sterile water for 10^−1^ dilution. The mixture was homogenized by vortexing to ensure uniform distribution of microbial cells. Subsequently, 100 μL of the diluted sample was plated onto various universal and selective culture media using sterile pipette tips. The sample was evenly distributed on the agar surface with the aid of a sterile microbial plate spreader, ensuring optimal colony isolation and growth.

To maximize the recovery of a diverse range of microorganisms, the following culture media were employed:

- Tryptic Soy Agar (TSA; Sigma Aldrich, Steinheim, Germany)—a general-purpose medium supporting the growth of a wide variety of bacteria,
- Schaedler Agar (Sigma Aldrich, Steinheim, Germany)—suitable for the cultivation of anaerobic and facultative anaerobic bacteria,
- Columbia Blood Agar Base (Oxoid—Thermo Scientific, Waltham, MA, USA) with 5% of sterile sheep blood (Graso BIOTECh, Starogard Gd., Poland)—used for the detection of hemolytic activity and growth of fastidious organisms,
- MILK Plate Count Agar Count plate (Graso BIOTECh, Starogard Gd., Poland)—designed for the enumeration of lactic acid bacteria and other dairy-associated microorganisms,
- LB Agar consisting of sodium chloride (P. P. H. “STANLAB” Sp. J., Lublin, Poland), yeast extract (Sigma Aldrich, Steinheim, Germany), tryptone (Sigma Aldrich, Steinheim, Germany) and agar (Sigma Aldrich, Steinheim, Germany)—a nutrient-rich medium suitable for general bacterial cultivation.

The culture media were in the form of ready-to-use powders.

The primary plates with the tested samples were incubated at both 37 °C ± 2 °C and 25 °C ± 2 °C to isolate a diverse range of microorganisms. Plates were monitored for up to 7 days to ensure the isolation of slow-growing microbial species. Culture conditions were changed through utilization of normal atmosphere and enriched in CO_2_. Upon initial colony formation, morphological analysis was performed to select distinct bacterial colonies. Using a sterile inoculating loop (1 μL), individual colonies were transferred to fresh culture plates to establish pure bacterial cultures. The secondary incubation followed the same temperature and atmospheric conditions as the primary incubation. However, the incubation time was 12 to 14 h. The growth time evaluation time was prepared, consisting of minimal detection and optimal culture time (from 12 h to 7 days).

### 3.3. Determination of Physicochemical Properties of Honey

#### 3.3.1. Color of Honey Samples

Each honey sample tested was dissolved (5 g of honey in 10 g of water to obtain a 50% solution) and then heated to 40 °C. The mixture was vortexed to achieve homogeneous dissolution of the ingredients. The color of the samples was determined by measuring the absorbance at λ = 635 nm using a UV-Vis Spectrophotometer NanoDrop 2000c (Thermo Fisher Scientific, Waltham, MA, USA). Measurements have been made in triplicate. The colors of the honeys were classified according to Pfund’s scale after converting the absorbance (Abs) values [20]

*mmPfund* = −38.70 + 371.39 × Abs; (1)

where *mmPfund* is the intensity of the honey color in the Pfund’s scale; Abs is the absorption of honey solution.

#### 3.3.2. pH Measurements

A total of 6 g of each honey was dissolved in 45 mL of water. Subsequently, the pH of the solution was measured using a pH-meter (CPC-501) with a glass electrode from Elmetron, Chorzów, Poland. Measurements were taken three times.

#### 3.3.3. MALDI-TOF/MS Identification

Isolated bacteria were identified using matrix-assisted laser desorption ionization time of flight mass spectrometry with a MALDI microflex^®^ LT/SH MALDI-TOF mass spectrometer featuring an N2 laser providing a 60 Hz repetition rate and the MALDI Biotyper 4.1 platform (Bruker Daltonics GmbH, Bremen, Germany). Bacteria samples were prepared following Bruker’s guidelines for the Extended Direct Transfer protocol. For this purpose, pure bacterial colonies were transferred directly by an inoculation loop onto an MBT Biotarget 96 IVD MALDI target (Bruker Daltonics GmbH, Bremen, Germany), air-dried, and covered with 1 µL of 70% formic acid. Then, 1 µL of the MALDI matrix solution—10 mg/mL α-cyano-4-hydroxycinnamic acid (HCCA; Bruker Daltonics GmbH, Bremen, Germany)—dissolved in a standard solvent solution (50% acetonitrile, 47.5% water, 2.5% trifluoroacetic acid) was coated. Due to the high prevalence of Gram-positive bacteria [66], the study initially also employed 2,5-Dihydroxybenzoic acid (DHB) matrix; however, unsatisfactory results led to the discontinuation and utilizing only HCCA. Two spots were prepared for each sample. The obtained mass spectra underwent a smoothing and baseline correction procedure using the flexControl program (Bruker Daltonics GmbH, Bremen, Germany).

## 4. Conclusions

The study highlights the key role of isolation and incubation conditions in the successful identification of environmental microorganisms from honey samples. By adjusting culture time and temperature, we can not only increase microbial diversity but also improve the precision of MALDI-TOF/MS identification, which is essential for detecting potential pathogens such as *Paenibacillus larvae*, a key agent of American foulbrood. Longer incubation increased microbial count, with organisms from 19 families identified. Temperature also affected identification precision, with optimal results for *Bacillus subtilis* subsp. *subtilis* and *Bacillus cereus* at 25 °C, and *Bacillus pumilus* at 37 °C, possibly reflecting temperature-dependent protein synthesis and endospore activity. MALDI-TOF/MS analysis also enabled the preliminary identification of bacteriocins that may inhibit pathogenic bacteria.

Statistical analysis showed no significant effect of pH on bacterial counts, but a slight increase in pH favored *Bacillus subtilis* subsp. *subtilis* presence. Honey type, rather than region of origin, significantly influenced bacterial profiles, with *Bacillaceae* and *Paenibacillaceae* families most commonly identified. This work opens the door to further research into the antimicrobial properties of bacteria isolated from honey, which could be crucial for developing new natural preservatives or treating bacterial infections, potentially supporting the ongoing fight against antibiotic-resistant pathogens.

## Figures and Tables

**Table 1:** List of investigated honeys with marked types and region of origin and physicochemical parameters.

Honey Type	Region				Pfund Scale		pH Value
Multifloral with spirulina 0.4%	Kuyavian–Pomeranian Voivodeship, Poland				584.37		3.54
Linden	Lower Silesian Voivodeship, Poland				6.27		3.48
Nectar-Honeydew	Lower Silesian Voivodeship, Poland				7.35		3.57
Multifloral	Lower Silesian Voivodeship, Poland				8.97		3.34
Buckwheat	Kuyavian–Pomeranian Voivodeship, Poland				36.44		3.21
Rapeseed	Kuyavian–Pomeranian Voivodeship, Poland				8.20		3.38
Multifloral	Kuyavian–Pomeranian Voivodeship, Poland				3.67		3.37
Phacelia	Masovian Voivodeship, Poland				3.39		3.33
Multifloral	Masovian Voivodeship, Poland				2.13		3.23
Multifloral	Masovian Voivodeship, Poland				7.50		3.10
Honeydew Cherry	Masovian Voivodeship, Poland				87.82		3.43
Multifloral	Masovian Voivodeship, Poland				1.11		3.48
Multifloral	Masovian Voivodeship, Poland				2.65		3.33
Multifloral	Masovian Voivodeship, Poland				1.78		3.42
Rapeseed	Lower Silesian Voivodeship, Poland				6.11		3.31
Linden	Lower Silesian Voivodeship, Poland				7.85		3.16
Acacia	Lower Silesian Voivodeship, Poland				1.36		3.24
Multifloral	Tuscany, Italy				9.09		3.25
Multifloral	Istanbul, Türkiye				0.82		3.44
Multifloral	Ankara, Türkiye				3.33		3.84
Multifloral	Masovian Voivodeship, Poland				18.62		4.43
Multifloral	Zakynthos, Greece				14.90		3.79
Honeydew–Forest	Saxony, Germany				302.36		3.82
Linden	Saxony, Germany				47.46		3.42
Sunflower	Saxony, Germany				97.85		3.35
Multifloral	Ankara, Türkiye				35.70		4.03
Multifloral	Blaj, Romania				125.95		3.34
Multifloral with lavender	Croatia				95.99		3.51
Multifloral	Bologna, Italy				98.22		3.29
Leatherwood	Tasmania				7.48		3.59
Clover	Tasmania				6.66		3.98
Linden	Lublin Voivodeship, Poland				65.66		3.60
Linden	Masovian Voivodeship, Poland				153.56		3.40
Multifloral	Masovian Voivodeship, Poland				225.36		3.27
Buckwheat	Podlaskie Voivodeship, Poland				269.80		3.12
Multifloral	Lublin Voivodeship, Poland				114.81		3.33
Rapeseed	Lublin Voivodeship, Poland				83.24		3.59
Buckwheat	Lower Silesian Voivodeship, Poland				525.81		3.16
Acacia	Lower Silesian Voivodeship, Poland				134.86		2.99
Linden	Lower Silesian Voivodeship, Poland				136.22		3.25
Multifloral	Cameroon, Africa				172.74		3.65
Grape ivy	Lower Silesia Voivodeship, Poland				351.51		3.26
-	Illinois, USA				31.37		4.02
Multifloral	France				163.83		3.48
Raspberry	Podlaskie Voivodeship, Poland				451.78		3.56
Buckwheat	Podlaskie Voivodeship, Poland				330.09		2.98
Linden	Podlaskie Voivodeship, Poland				103.54		3.68
Melilot-cornflower	Pomeranian Voivodeship, Poland				129.17		3.56
Phacelia	Kuyavian–Pomeranian Voivodeship, Poland				65.54		3.21
Blueberry	Kuyavian–Pomeranian Voivodeship, Poland				50.80		3.45
Hawthorn	Warmian–Masurian Voivodeship, Poland				88.44		3.38
Raspberry	Lower Silesian Voivodeship, Poland				149.72		3.71
White sweet clover	Subcarpathian Voivodeship, Poland				188.84		3.50
Linden	Łódź Voivodeship, Poland				236.50		3.14
Multifloral	Lower Silesian Voivodeship, Poland				213.72		3.28
Fennel	Podlaskie Voivodeship, Poland				354.60		3.29
Multifloral	Lisbona, Portugal				95.99		3.45
Multifloral	Lower Silesian Voivodeship, Poland				132.51		3.58
Chinese acacia, fennel	England				32.48		3.44
Multifloral	Israel				210.26		3.47
Multifloral	Lithuania				131.52		3.47
-	Kuyavian–Pomeranian Voivodeship, Poland				43.87		3.39
Cinnamon	Poland				1169.31		3.10
Raspberry	Poland				967.52		3.13
Orange	Poland				261.38		3.27
Linden	Germany				101.19		3.69
Multifloral	Sweden				134.74		3.58
Multifloral	Armenia				86.33		3.27
Linden	Armenia				12.43		3.34
-	Germany				83.49		3.31
Multifloral	Czechia				130.78		3.90
Multifloral	Valencia, Spain				90.42		3.63
-	Ukraine				62.19		3.84
Pfund Scale	Water White <9	Extra White 9–17	White 18–34	Extra Light Amber 35–50	Light Amber 51–85	Amber 86–114	Dark Amber >114

## Data Availability

The original contributions presented in this study are included in the article. Further inquiries can be directed to the corresponding authors.
